# Changes in kynurenine metabolites in the gray and white matter of the dorsolateral prefrontal cortex of individuals affected by schizophrenia

**DOI:** 10.1038/s41537-024-00447-3

**Published:** 2024-02-27

**Authors:** Nico Antenucci, Giovanna D’Errico, Francesco Fazio, Ferdinando Nicoletti, Valeria Bruno, Giuseppe Battaglia

**Affiliations:** 1 Department of Physiology and Pharmacology, Sapienza University, Rome, Italy; 2 IRCCS Neuromed, Pozzilli, Italy; 3 Present Address: Department of Pharmacology and Neuroscience, Texas Tech University Health Sciences Center, Lubbock, TX USA; 4 Present Address: Department of Psychiatry, University of California San Diego, La Jolla, CA USA

**Keywords:** Schizophrenia, Schizophrenia

## Abstract

Alterations in the kynurenine pathway of tryptophan metabolism have been implicated in the pathophysiology of schizophrenia. Here, we performed an in-depth analysis of all metabolites of the kynurenine pathway, i.e., tryptophan (TRY), kynurenic acid (KYNA), L-kynurenine (KYN), 3-hydroxykynurenine (3-HK), anthranylic acid (ANA), 3-hydroxyanthranylic acid (3-HANA), xanthurenic acid (XA) and quinolinic acid (QUINA), in postmortem samples of the dorsolateral prefrontal cortex (DLPFC, Brodmann area 46, 9) of individuals affected by schizophrenia and non-schizophrenic controls. The analysis was carried out in the gray and white matter. Levels of KYN, 3-HK, ANA, and 3-HANA were significantly increased in both the gray and white matter of the DLPFC of individuals affected by schizophrenia, whereas levels of TRY, KYNA, and QUINA were increased exclusively in the white matter and remained unchanged in the gray matter. These increases in kynurenine metabolites did not correlate with age, sex, duration of the disease, and duration and type of antipsychotic medication. These findings suggest that the two major branches of the kynurenine pathway, i.e., the transamination of KYN into KYNA, and hydroxylation of KYN into 3-HK are activated in the white matter of individuals affected by schizophrenia, perhaps as a result of neuroinflammation, and support the evidence that abnormalities of the white matter are consistenly associated with schizophrenia.

## Introduction

The efficacy of current antipsychotics is limited in improving negative and cognitive symptoms of schizophrenia^1,2^. Type-2 and −4 muscarinic receptors and type-1 trace amine receptors are examples of targets for new therapeutic agents that showed efficacy in clinical studies^3,4^. To our knowledge, there are no drugs that slow the progression of schizophrenia, which is driven by neuroinflammation and loss of gray and white matter occurring since the early, preclinical phase of the disease^5^. The kynurenine pathway of tryptophan metabolism^6–8^ is a biochemical *trait d’union* between neuroinflammation and abnormalities in excitatory neurotransmission, and has been implicated in the pathophysiology of schizophrenia^8–12^. The first step of the pathway is the opening of the pyrrole moiety of L-tryptophan to yield formyl-kynurenine, which is spontaneously converted into L-kynurenine (KYN). The reaction is catalyzed by type-1 and −2 indoleamine 2,3-dioxygenase (IDO1 and −2) and tryptophan 2,3-dioxygenase (TDO). IDO is induced by interferon-γ, interleukin-1β, and other proinflammatory cytokines in many organs, including the CNS^13–18^. KYN is hydroxylated into 3-hydroxykynurenine (3-HK) by kynurenine monooxygenase (KMO), or, alternatively, is transaminated into kynurenic acid (KYNA) by kynurenine aminotransferase (KAT). 3-HK is sequentially transformed into 3-hydroxyanthranilic acid (3-HANA) and quinolinic acid (QUINA), or, alternatively, is transminated by KAT into xanthurenic acid (XA)^19,20^. Some kynurenine metabolites interact with membrane receptors and modulate neurotransmission in the CNS. KYNA is an antagonist at the glycine site of N-methyl-D-aspartate (NMDA) receptors^21^, but can also block other glutamate receptor subtypes at high concentrations^22^. In addition, KYNA antagonizes homopentameric neuronal nicotinic receptors containing the α7 subunit^23^, and interacts with GPR35 an HCAR3 G-protein coupled receptors^24,25^. In contrast, QUINA binds to the GluN2 subunits of NMDA receptors acting as an orthosteric agonist^26,27^. Xanthurenic acid modulates the function of mGlu2 metabotropic glutamate receptors, whereas cinnabarinic acid, a by-product of the kynurenine pathway formed by the condensation of two molecules of 3-HANA, is a weak orthosteric agonist of mGlu4 receptors^28–31^.

CSF and brain KYNA levels are consistently increased in individuals affected by schizophrenia^32,33^, owing to a reduced activity of KMO^34^. The increase in KYNA:QUINA ratio, which is expected to restrain the endogenous activation of NMDA receptors, is consistent with the hypoglutamatergic hypothesis of schizophrenia^35–38^. We found a reduction in the levels of cinnabarinic acid in the prefrontal cortex of individuals affected by schizophrenia^31^. This reduction might be also linked to the pathophysiology of schizophrenia because cinnabarinic acid displayed potent antipsychotic-like activity in animal models^31^. However, the connection between the kynurenine pathway and schizophrenia may not be restricted to KYNA and QUINA.

Whether changes in other kynurenine metabolites occur in brain tissue of individuals affected by schizophrenia is less clear. Here, we aim to address the existing gaps in the literature pertaining to the comprehensive analysis of kynurenine pathway metabolites in postmortem samples of the dorsolateral prefrontal cortex (Broadmann area 46, 9) of individuals affected by schizophrenia and non-schizophrenic controls. By performing a simultaneous measurement of all metabolites of the kynurenine pathway in optically dissected gray and white matter, our research not only contributes to a deeper understanding of the neurobiological underpinnings of schizophrenia, but may also pave the way to novel therapeutic strategies.

## Results

We measured levels of kynurenine metabolites in the gray and white matter dissected from the DLPFC of individuals affected by schizophrenia (SCZ, 14 males and 7 females, age: 25–61 years; mean ± SEM = 49 ± 2.3) and non-schizophrenic controls (CTRL, 20 males and 4 females, age: 36–63 years; mean ± SEM = 55 ± 1.5). There was no statistical difference between the ages of controls and individuals affected by schizophrenia (Mann-Whitney Rank Sum Test). Information on drug treatment was available for 15 subjects of the CTRL group and 16 subjects of the SCZ group. Individuals affected by SCZ had been treated with either first- or second-generation antipsychotics (*n* = 2 or 3 and 9, respectively), in some cases combined with valproate or lithium. Duration of antipsychotic medication ranged between 1 and 30 years. Subjects of both groups had also been treated with other types of drugs, such as drugs for cardiovascular disorders, anti-diabetic drugs, and opioid analgesics (Tables 1 and 2). Neuropathological analysis showed signs of amyloid and tau pathology, and cerebrovascular pathology in some samples of both groups. One individual of the SCZ group was also affected by multiple sclerosis (Tables 1 and 2). All available data provided by the biobank relative to controls and subjects affected by schizophrenia, and neuropathological data of brain samples are shown in Tables 1 and 2.

**Table 1:** Information on sex, age at death, postmortem interval (PMI), drug treatment, duration of prescription, and neuropathological characteristics in subjects of the control group.

ANONYMOUS #	Diagnosis	Duration of Diagnosis	Duration of Prescription	Prescription	Sex	Age	PMI	Neuropathology
AN19744	Control	N/A	INR	Aspirin, Oxycontin	F	59	13.08	Remote infarct, frontal operculum and insular cortex, and corona radiata lateral to head of caudate nucleus; neurofibrillary tangles, Braak stage I.
AN11537	Control	N/A	INR	Prednisone 50 mg sid, Pentoxil 400 mg bid, Vitamin E 600 bid, Altace 5 mg sid, Famotidine 20 mg prn, Zofran 4 mg prn, Percocet 5/325 mg prn, Dilaudid 2 mg prn	F	60	12.53	Mild atherosclerosis and arteriosclerosis, cerebral arteries.
AN06102	Control	N/A	3 yr/INR	Atenolol/Insulin, Iron Vitamin, Neurontin, Wellbutrin	F	53	16.62	No neuropathological abnormality; cerebral arteriolosclerosis, moderate; ischemic/hypoxic encephalopathy
AN15240	Control	N/A	INR	Lexapro, Lorazepam	F	36	18.08	No diagnostic abnormality
AN15708	Control	N/A	INR	INR	M	40	16.6	There is no diagnostic abnormality recognized.
AN00452	Control	N/A	15 yrs	Angina and pain meds	M	58	17.5	Normal brain.
AN05671	Control	N/A	INR	Isosorbide, Plavix, Lipitor, Folic Acid, Vitamin E	M	58	15.5	No diagnostic abnormality.
AN12240	Control	N/A	INR	INR	M	51	4.75	No diagnostic abnormality.
AN01275	Control	N/A	INR	Oral meds for diabetes, Vitamins	M	61	10.08	Arteriolosclerosis and mild edema, cerebral white matter; neurofibrillary tangles, Braak stage I.
AN10090	Control	N/A	5 yr/10 yr	Lisinopril/Glyburide, Insulin	M	52	13.12	Mild cerebral amyloid angiopathy.
AN12699	Control	N/A	INR	High blood pressure meds, Vitamins	M	55	11.02	No neurapathological abnormality; acute hypoxic/ischemic encephalopathy; cerebral arteriolosclerasis, mild to moderate.
AN01254	Control	N/A	INR	INR	M	59	17.28	Neurofibrillary tangles, Braak stage I. Comment: Braak staging of neurafibrillary degeneration is based on a scale of I-VI. Stage I is without clinical significance.
AN16799	Control	N/A	INR	Metoprolol, Aspirin, Plavix, Lipitor	M	43	14.68	Rare neurafibrillary tangles, nucleus basalis of Meynert. Comment: neurofibrillary tangles are usually staged on a scale of I-VI (Braak and Braak), but is unclear whether a few tangles in the nucleus basalis of Meynert qualify as stage I.
AN13797	Control	N/A	2 yr/INR	High blood pressure meds/Naproxen, Albuterol inhaler as needed	M	61	17	Cerebravascular disease, with mild atherasclerasis and a small lacune in the putamen.
AN03233	Control	N/A	INR	Glucophage, High blood pressure meds, High cholesterol meds	M	62	18.33	Neurafibrillary tangles, Braak stage I; cerebral edema (brain swelling), mild; atherosclerosis, cerebral arteries. Comment: brain swelling is a common nonspecific phenomenon of the terminal premortem period.
AN15472	Control	N/A	INR	MSM, Glucosamine, Ibuprofen	M	57	18.15	Neurofibrillary tangles, Braak stage I; amyloid angiopathy, mild; minor acute subarachnoid hemorrhage. Comment: these pathological findings are likely to have been asymptomatic. The hemorrhage is a phenomenon of the terminal premortem course.
AN02896	Control	N/A	8 months/INR	Lipitor/Glucosamine, Naproxen	M	55	14.5	Neurofibrillary degeneration, Braak stage I; brain swelling with tentorial notching of ventral uncus (brain weight 1450 grams). Comment: Braak I is common above age 50 and is asymptomatic. Brain swelling is a common nonspecific phenomenon of the terminal premortem period.
AN13041	Control	N/A	INR	Lisinopril, Lasix, Flomax, Aspirin, Albuterol, Metolazone	M	63	17.67	Cerebrovascular disease, with mild atherosclerosis of middle cerebral artery, moderate arteriosclerosis of arteries of deep frontal white matter, and an infarct in the caudal inferior frontal gyrus; neurofibrillary tangles, Braak stage I. Comment: the frontal infarct was at the intermediate stage, was immediately rostral to Broca’s area, and was undoubtedly symptomatic. Braak staging of neurofibrillary degeneration is based on a scale of I-VI. Stage I is common above age 50 and is asymptomatic.
AN02315	Control	N/A	INR	INR	M	55	17.8	Mild arteriosclerosis. Comment: arteriosclerosis usually reflects a history of hypertension.
AN01235	Control	N/A	INR	Cardia, Lisinopril, Loratadine	M	52	7.83	Neurofibrillary tangles, Braak stage I. Sparse leptomeningeal mononuclear infiltrate. Comment: a leptomeningeal infiltrate is consistent with a chronic systemic infectious or leukopraliferative process, but the sparsity of the cells suggests a disorder with only minor clinicaI expression or the remnant of a remote event.
AN13021	Control	N/A	INR	Lisinopril 10 mg, Lasix 160 mg, Potassium 20 mcg, Metoprolol 50 mg	M	55	23.18	No diagnostic abnormality.
AN13112	Control	N/A	INR	Vytorin	M	63	17.92	Neurofibrillary degeneration, Braak stage II, with neuritic neocortical amyloid plaques and mild amyloid angiopathy; atherosclerosis and arteriosclerosis. Comment: arteriosclerosis usually reflects a history of hypertension.
AN19145	Control	N/A	INR	Plavix, B12, Gabapentin, Glipizide	M	64	14.33	Mild arteriosclerosis. Neurofibrillary degeneration, Braak stage I, with rare to sparse non-neuritic neocortical amyloid plaques.
AN04134	Control	N/A	INR	INR	M	56	17.32	Small vessel cerebrovascular disease with atherosclerosis, mild arteriosclerosis and arteriolosclerosis, two microinfarcts in the lateral segment of the globus pallidus, and one in the putamen; neurofibrillary degeneration, Braak stage I.

**Table 2:** Information on sex, age at death, postmortem interval (PMI), RNA integrity number (RIN), pH, duration of diagnosis, duration of prescription, prescription, agonal duration, presence of fever and infection during the agonal state, hypoxia duration of the agonal state, antipsychotic medication at the time of death, duration of the med-free interval prior to death, and neuropathological characteristics in patients affected by schizophrenia.

ANONYMOUS #	Diagnosis	Duration of Diagnosis	Duration of Prescription	Prescription	Sex	Age	PMI	RIN	pH	Agonal duration (from Hardy et al 1985) & Agonal duration (from Tomika 2004)	Fever	Infection	Hypoxia duration of the agonal state	Antipsychotic medication at the time of death	Duration of the med-free interval prior to death	Neuropathology
AN09422	Schizophrenia	INR	INR	INR	M	62	6.1	3.2	/	/	/	/	No	/	/	lnfarcts (2), old, right thalamus (dorsomedian nucleus [microscopicaI]; and lateral posterior-ventral posterolateral nuclei [0.4 × 0.2 × 0.2 cm])
AN10924	Schizophrenia	INR	INR	INR	M	44	19	/	/	3: Intermediate, 1–24 h; 1: Intermediate and slow (1–24 h)	/	Yes	/	/	/	There is no diagnostic abnormality recognized
AN04704	Schizophrenia	MDD 15 yr; S 1 yr	1 yr	Trazodone, Prozac, Olanzapine	M	42	18.1	/	/	Fast death natural causes <1 h; 0: Fast deaths/terminal phases <1 h	No	No	Yes	/	/	Cerebral edema and diffuse purple discoloration consistent with acute monoxide poisoning; encephalopathy of hypoxic-ischemic type, acute, mild.
AN04112	Schizophrenia	27	1 yr/2 yrs	Zyprexa 10 mg/Depakote 500 mg x4/day, Haldol shots	M	46	18.5	/	/	N/A not known: COD is Sepsis	No	Yes	No	/	/	There is no diagnostic abnormality recognized.
AN00989	Schizophrenia	INR	INR	INR	M	47	19.25	/	/	4: Slow death (e.g., carcinoma) > 24 h; 1: Intermediate and slow (1–24 h)	No	No	No	Yes, prescribed antipsychotics at time of death. Haldol	N/A: antipsychotics prescribed at time of death	Metastatic (adeno) carcinoma involving cerebellar leptomeninges; and cerebral, and cerebellar parenchyma; hippocampal sclerosis; infarct, old, small, cortical, superior parietal lobule; infarct, old, small, putamen
AN07652	Schizophrenia	INR	INR	INR	M	61	19.9	/	/	N/A not known: COD is Sepsis	No	Yes	No	Yes, prescribed antipsychotics at time of death. Stelazine, Mellaril, Haldol, Clozamil, Risperidone, Atarax	N/A: antipsychotics prescribed at time of death	Cerebral arteriolosclerosis, moderate; resolving cortical microinfarcts, frontal cortex; acute infarct, frontal white matter
AN17681	Schizophrenia	INR	INR	INR	M	31	20	/	/	N/A not known: COD Pulmonary Embolism	No	No	Yes	Yes, prescribed antipsychotics at time of death. Prolixin, Clozail, Seroquel, Serentil, Chlorpromazine, Haldol, Zyprexa, Loxapine Succinate, Navane, Loxitane, Risperdal, Loxapine	N/A: antipsychotics prescribed at time of death	No neuropathological diagnostic abnormality; cerebral arteriolosclerosis, moderate to severe, with Etat Criblae
AN14320	Schizophrenia	38	30 yrs/10 yrs	Prolixin/Risperdal	F	61	14.08	/	/	4: Slow death (e.g., metastatic endometrial cancer) > 24 h; 1: Intermediate and slow (1–24 h)	No	Yes	No	Yes, prescribed antipsychotics at time of death. Thorazine, Prolixin, Risperdal, Zyprexa, Luvox	N/A: antipsychotics prescribed at time of death	Mild neuron loss, inferior olive, dentate nucleus, Purkinje cells of vermis; neurofibrillary tangles, Braak stage III. Comment: the olivo-dentato-cerebellar changes are unexplained, but are mild. Neurofibrillary tangles without senile plaques do not indicate Alzheimer’s disease.
AN04402	Schizophrenia	40	INR	Klonopin 1 mg x 2/day, Clozaril 150 mg x 2/day, Citracal 1500/200/day, Paxil 20 mg/day, Senokot 51 × 2/day, Hydrochlorothiazide, ASA, Tylenol as needed	F	61	11	/	/	2: Fast death natural causes <1 h; 0: Fast deaths/terminal phases <1 h	No	No	Yes	Yes, prescribed antipsychotics at time of death. Thorazine, Clozapine, Paxil, Haldol, Stelazine	N/A: antipsychotics prescribed at time of death	No diagnostic abnormalities were observed. Comment: mild myelin pallor with arteriaI medial hyperplasia was seen in the deep white matter, a common and nonspecific finding.
AN17781	Schizophrenia	7	4 yrs	Aspirin, OTC meds, Sudafed, Zyprexa (noncompliant)	F	40	18.4	/	/	N/A not known: COD is suicide of unknown manner	/	/	/	/	/	No diagnostic abnormality.
AN09613	Schizophrenia	46	INR	Divalproex 500 mg x 2/day, Levothyroxine 0.125 mg, Olanzapine 10 mg, Lorazepam 0.5 mg as needed	F	56	18.72	4.4	5.98	Fast death natural causes <1 h; 0: Fast deaths/terminal phases < 1 h	No	No	No	Yes, prescribed antipsychotics at time of death. Lithium, Depakote, Zyprexa, Haldol, Risperidone, Invega, Clozaril	N/A: antipsychotics prescribed at time of death. Providor notes medication appears to be of significant benefit to her	White matter microinfarcts, occipital lobe; vascular mineralization, basal ganglia.
AN09353	Schizophrenia	25	2 yrs	Seroquel 800 mg, Zoloft and Trilafon 125 mg	M	47	17.75	/	/	4: Slow death (e.g., cancer) > 24 h; 1: Intermediate and slow (1–24 h)	No	No	No	Yes, prescribed antipsychotics at time of death. Triavil, Chlorpromazine, Haldol, Mellaril, Perphenazine, Zyprexa, Seroquel	N/A: antipsychotics prescribed at time of death	Senile plaques, diffuse, basal temporal lobe; mild acute hippocampal neuron loss in field CA4. Comment: diffuse senile plaques alone, at sparse to moderate density, would not be associated with any clinicaI expression. This brain corresponds to the Braaks’ stage A for amyloid deposition (scale: A-C), and stage 0 for neurofibrillary degeneration (scale I-VI). The mild, acute hippocampal field CA4 neuron loss is, a common, nonspecific finding in the postmortem brain.
AN19277	Schizophrenia	INR	INR	INR	M	46	17.67	/	/	2: Fast death natural causes <1 h; 0: Fast deaths/terminal phases <1 h	No	No	Yes	Yes, prescribed antipsychotics at time of death. Zyprexa	N/A: antipsychotics prescribed at time of death. When asked about effectiveness, family responded “yes?” in questionnaire	Remote frontal cortical and white matter lnfarct; multiple cavernous angiomas; cerebral arteriolosclerosis, mild to moderate; acute hypoxic/anoxic encephalopathy. Comments: neurofibrillary tangles in nucleus basalis of Mynert may be secondary to the large infarct in the frontal lobe.
AN02816	Schizophrenia	22	22 yrs	Thioridazine 100 mg/day, Ibuprofen	F	52	16.12	/	/	4: Slow death (e.g., cancer) > 24 h; 1: Intermediate and slow (1–24 h)	No	No	No	/	/	No diagnostic abnormality
AN15934	Schizophrenia	15	13 yrs/8 yrs/0.5 yrs	Clozapine/Prozac/Seroquel and Abilify	M	36	17.97	/	/	3: Intermediate, 1–24 h: 1: Intermediate and slow (1–24 h)	No	No	Yes	Yes, prescribed antipsychotics at time of death. Clozaril, Clozapine, Seroquel, Abilify, Depakote	N/A: antipsychotics prescribed at time of death. Family notes they were partially effective	No neuropathological abnormality; acute hypoxic/anoxic encephalopathy
AN12157	Schizophrenia	43	2 yrs/ 0.5 yrs	Acetonel 35 mg wkly, Valproic acid 375 mg x2/day, Celexa 10 mg/day/Norvasc, Lasix	M	62	10.75	/	/	4: Slow death (e.g., cancer) > 24 h; 1: Intermediate and slow (1–24 h)	No	Yes	No	Yes, prescribed antipsychotics at time of death. Clozaril	N/A: antipsychotics prescribed at time of death	Moderate arteriosclerosis, and mild myelin pallor, deep frontal white matter; microinfarct, putamen
AN02314	Schizophrenia	23	INR	Lithium 600 mg x 2/day, Risperdal, Trazodone 50 mg/day, Spiriva. Sumtjrpod 0.175 mg/day, Lasix, Lipitor 40 mg/day, ASA, Theophylline 300 mg 2x/day, Prilosec, Insulin	M	44	8.5	7.4	/	2: Fast death natural causes <1 h; 0: Fast deaths/terminal phases <1 h	No	Unk	No	/	/	Severe axon loss, optic tract; neurofibrillary degeneration, Braak stage I; mild autolytic changes. Comment: no clinicaI history of blindness is presently available to correlate with the optic tract degeneration. Braak staging of neurofibrillary degeneration is difficult because the entorhinal cortex is not included in the specimen. The absence of tangles in the tissue examined except for one pretangle in the nucleus basalis suggests a likely stage of I (on a scale of I-VI). Autolytic change in the tissue is a postmortem phenomenon, commonly observed in autopsied brain tissue.
AN13227	Schizophrenia	40	3–4 yrs	Metoprolol 50 mg x 2/day, Seroquel 200 mg x 4/day, Lithium, Haloperidol 20 mg/day	F	60	17.13	/	/	/	/	/	/	/	/	Arteriosclerosis; single neurofibrillary tangle, locus coeruleus, and mild amyloid angiopathy. Comment: a Braak stage of neurofibrillary degeneration cannot be assigned because the hippocampus and entorhinal area are unavailable. A stage higher than I or possibly early Il is highly unlikely based on the absence of tangles in the nucleus basalis of Meynert. Amyloid angiopathy is a frequent accompaniment to neurofibrillary degeneration, and is asymptomatic.
AN11287	Schizophrenia	32	INR	Benztropine 0.5 mg x 2/day, Clozarail 10 mg, Depakote 125 mg x3/day, Fluphenazine HCl 10 mg x 2/day	M	49	8.17	/	/	4: Slow death (e.g., cancer) > 24 h; 1: Intermediate and slow (1–24 h)	Unk	No	Unk	/	/	Necrotic gigantocellular tumor in pia with minor invasion of occipital cortex and cerebellar cortex (history of metastatic lung carcinoma and chemotherapy); Arteriosclerosis; Neurofibrillary tangles, Braak stage I
AN17799	Schizophrenia	29	15	Clozaril, Navane, Nystatin, Cogentin	F	56	10.5	6	/	4: Slow death (e.g., cancer) > 24 h; 1: Intermediate and slow (1–24 h)	unk	No	unk	Yes, prescribed antipsychotics at time of death. Zyprexa, Thorazine, Celexa	N/A: antipsychotics prescribed at time of death	Arteriosclerosis
AN10994	Schizophrenia	7	3 yrs	Abilify 400 mg intramuscular monthly; Ativan 0.5 2x/day. Doxepin 10 mg/day for last 8 months. During last 3 days was administered Unasyn, Pepcid, Haparin, Fentanyl, Propofol, Versed	M	25	17.09	/	6.17	fentanyl OD, anoxic brain injury, intubated for unknown duration	/	/	/	N/A no medical records. Tocixology reports lorazepam, midazolam, delta-9-THC, fentanyl, norfentanyl	N/A no medical records	Acute ischemie injury. Comment: there is acute ischemic change throughout the brain, with preservation of cells (e.g., in the cerebellar granule cell layer), except in the most affected areas such as cerebral cortex and basal ganglia

**Fig. 1:**
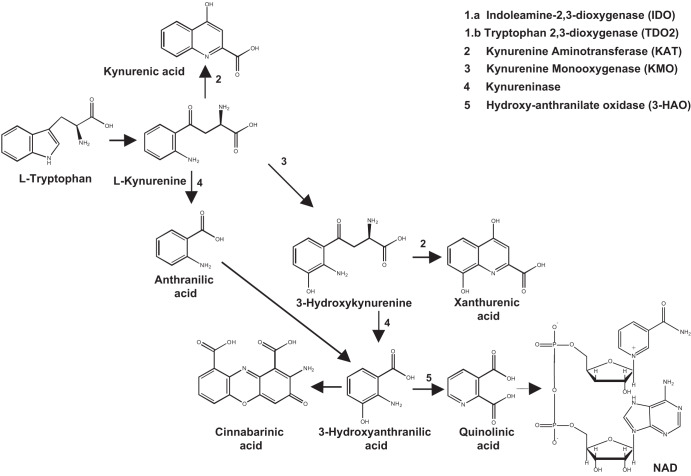
The kynurenine pathway. Metabolites and enzymes involved are shown.

As the rate of degradation of kynurenine metabolites in human brain tissue is unknown, it was important to examine the possible correlation between levels of all kynurenine metabolites and the postmortem interval (PMI), i.e., the time elapsed between death and tissue removal. PMI ranged from 4.75 and 23.18 h in samples of both groups. No significant correlation was found between any kynurenine metabolite and PMI in both the gray and white matter of the DLPFC (Fig. 1S and ref. ^31^). Covariance analysis confirmed the lack of correlation between PMI, and all kynurenine metabolites in CTRL and SCZ samples with the exception of KYNA levels, which were inversily correlated to PMI in the gray matter (Pearson coefficient = −0.33, *p* = 0.035).

### Changes in the levels of kynurenine metabolites in the DLPFC gray matter of individuals affected by schizophrenia

**Fig. 2:**
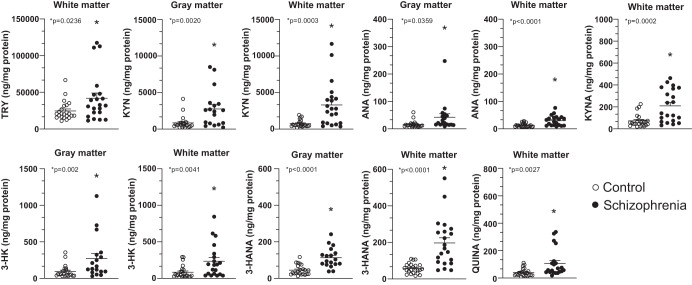
Cumulative endogenous levels of kynurenine pathway metabolites in both gray and white matter of DLPFC in control subjects (*n* = 24) and patients affected by schizophrenia (*n* = 21). *p < 0.05 (Student’s *t* test) vs. control subjects. Grubbs’s test was performed once and one sample for each group was excluded as an outlier, when the case.

**Fig. 3:**
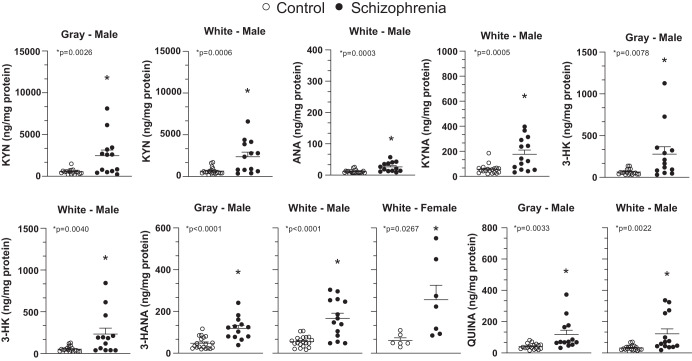
Endogenous levels of kynurenine pathway metabolites in gray and white matter of males and females in the DLPFC of control subjects and patients affected by schizophrenia. *p < 0.05 (Student’s *t* test) vs. control subjects. The control group is composed of 20 males and 4 females whereas the group of patients affected by schizophrenia is composed of 14 males and 7 females. Grubbs’s test was performed once and one sample for each group was excluded as an outlier, when the case.

**Fig. 4:**
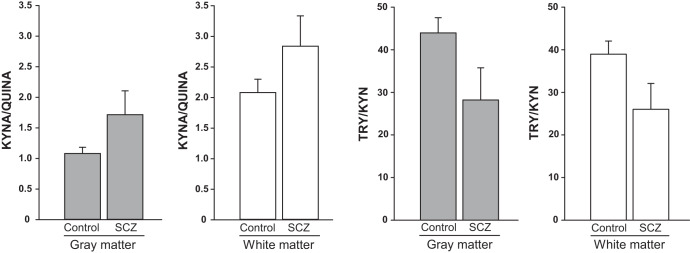
The KYNA:QUINA and TRY:KYN ratios are modified in the DLPFC of individuals affected by schizophrenia. The KYNA:QUINA ratio was increased by 55% and the TRY:KYN ratio was reduced by 36% in the gray matter of the SCZ group. The KYNA:QUINA ratio was increased by 34% and the TRY:KYN ratio was reduced by 33% in the white matter of the SCZ group.

Schematic representation of the kynurenine pathway is shown in the Fig. 1. Levels of KYN, 3-HK, ANA, and 3-HANA were significantly increased in the DLPFC gray matter of individuals affected by SCZ with respect to non-schizophrenic CTRL, whereas levels of TRY, KYNA, QUINA, and XA were unchanged (Figs. 2 and 2S). The increase in KYN, 3-HK, and 3-HANA was also statistically significant when the analysis was restricted to males of the SCZ and CTRL groups (Fig. 3). The analysis of female subjects was biased by the low number of samples in both groups (Fig. 3). We measured the ratio between TRY and KYN (a low ratio is indicative of the activation of the kynurenine pathway), and the ratio between KYNA and QUINA (a higher ratio is consistently observed in individuals affected by SCZ) (see Introduction and references therein). There was no significant difference between CTRL and SCZ in both the TRY:KYN and KYN:QUINA ratios (Fig. 4). However, the TRY:KYN ratio was reduced by 36%, and the KYNA:QUINA ratio increased by 55% in the gray matter of the SCZ group (Fig. 4).

### Increased levels of all kynurenine metabolites in the DLPFC white matter of individuals affected by schizophrenia

Levels of TRY and all kynurenine metabolites, with the exception of XA, were significantly increased in the DLPFC white matter of the SCZ group, and the increase in KYN, 3-HK, 3-HANA, and QUINA remained statistically significant when the analysis was restricted to males of both groups (Fig. 3).

Similarly to what observed in the gray matter, there was no significant difference between CTRL and SCZ in both the TRY:KYN and KYN:QUINA ratios (Fig. 4). However, the TRY:KYN ratio was reduced by 33%, and the KYNA:QUINA ratio was increased by 34% in the white matter of the SCZ group (Fig. 4).

### Correlation analysis between levels of kynurenine metabolites in the gray or white matter and age or duration of antipsychotic medication

**Fig. 5:**
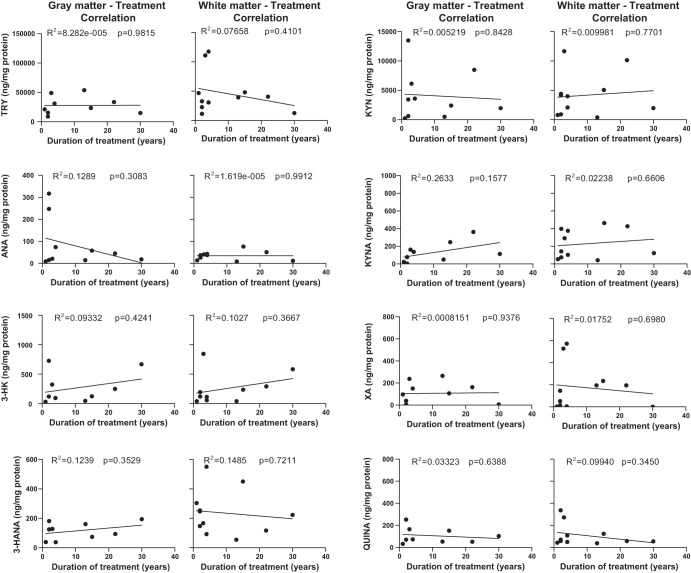
Lack of correlation between levels of TRY and kynurenine metabolites and the duration of disease. Correlation analysis between kynurenine metabolite levels in the gray and white matter and duration of antipsychotic medication.

**Fig. 6:**
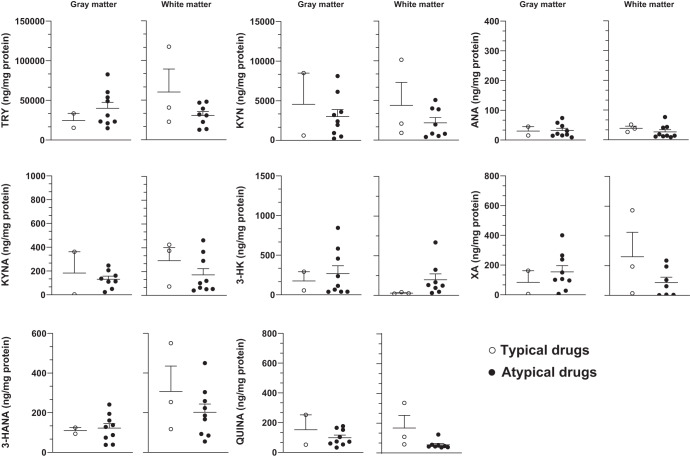
Lack of correlation between levels of kynurenine metabolites and the type of antipsychotic medication. Correlation analysis between kynurenine metabolite levels in the gray and white matter and duration of typical and atypical antipsychotic drugs.

There was no significant correlation between levels of TRY or kynurenine metabolites in the gray or white matter and age in either the CTRL or the SCZ group (Fig. 3S). In addition, in the SCZ group, there was no correlation between levels of TRY or any kynurenine metabolite and the duration (Fig. 5) and type of antipsychotic medication (Fig. 6). Covariance analysis confirmed the lack of correlation between age and kynurenine metabolites. There was also no correlation between sex and kynurenine metabolites in the gray matter. In contrast levels of 3-HANA (Pearson coefficient = 0.326, *p* = 0.028), ANA (Pearson coefficient = 0.401, *p* = 0.07), and KYNA (Pearson coefficient = 0.421, *p* = 0.004) in the white matter showed a significant correlation with sex, being greater in females.

## Discussion

The kynurenine pathway meets the requirement to play a key role in the pathophysiology of psychotic disorders because it regulates energy metabolism (in the form of nicotinamide adenine dinucleotide), the immune system, and excitatory neurotransmission^39^. As KYNA is the only known NMDA receptor antagonist, it was postulated that an increased production of KYNA might be causally related to schizophrenia. It is consistent with this hypothesis that experimentally induced increases in CNS KYNA levels causes a schizophrenia-like phenotype in experimental animals^40^, and KYNA levels are increased in the postmortem brain and CSF of individuals affected by schizophrenia^41–43^. However, how the other kynurenine metabolites behave in the brain of individuals affected by schizophrenia is less clear. Robert Schwarcz and his associates found that the activity of KMO and 3-hydroxyanthranilic acid dioxygenase (3-HAO, the enzyme that transforms 3-HANA into QUINA) was reduced in the prefrontal cortex (Brodmann areas 9 and 10) of individuals affected by schizophrenia, whereas activities of kynureninase, kynurenine aminotransferase II, and quinolinic acid phosphoribosyltransferase were unchanged. This was associated with an increase in KYNA levels and suggested an increased KYNA:QUINA ratio in schizophrenia^11^. Increases in KYN levels and TDO2 expession were found in the prefrontal cortex and anterior cingulate cortex of individuals affected by schizoprenia^44,45^. More recently, Kindler et al. 2020^46^ found that KYNA levels, the KYN:TRY ratio, and the transcripts of TDO and KATI/II were increased in the prefrontal cortex of a “high cytokine schizophrenia subgroup” identified by measurements of proinflammatory cytokine mRNA, and there was a positive correlation between KAT and glial fibrillary acidic protein mRNA. This suggests that central neuroinflammation causes the activation of the kynurenine pathway in schizophrenia, and that the increase in KYNA levels is secondary to reactive gliosis. The conclusion of a systematic review on peripheral and central kynurenine metabolites in psychiatric disorders was that brain/CSF KYNA levels are increased, whereas metabolites of the KMO-driven branch of the kynurenine pathway (e.g., 3-HK, XA, and QUINA) are unchanged in schizophrenia^47^.

Our results diverge from this conclusion. We found that levels of all kynurenine metabolites, with the exception of XA, were increased in the white matter, and levels of KYN, 3-HK, ANA, and 3-HANA were increased in the gray matter of the DLPFC of individuals affected by schizophenia. The homogenous activation of the kynurenine pathway in the white matter is in line with a large body of evidence suggesting that white matter abnomalities are associated with schizophrenia^48–54^. Fractional anisotropy MI analysis exploring age-related white matter trajectories in a large cohort of patients affected by schizophrenia and healthy controls demonstrates that white matter is affected in all stages of schizophrenia in a tract-specific manner since the early phases of myelin maturation^50^. It is believed that inflammation contributes to the pathophysiology of white matter damage in schizophrenia^55–57^. If so, the white matter might represent a preferential site of activation of the kynurenine pathway, explaining our data. Activation of the kynurenine pathway in the white matter of schizophrenic patients might represent a compensatory mechanism aimed at restraining immune activation and neuroinflammation^58–62^. The increase in the KYNA:QUINA ratio found in the white matter of individuals affected by schizophenia might limit the endogenous activation of NMDA receptors expressed by oligodendrocytes, thus limiting the excitotoxic component of myelin damage^63–66^. It is important to note that the between-subject differences in the abundance of glia cells, particularly astrocytes and microglia, could potentially contribute to the measured variations in kynurenine metabolite concentrations. Future research is needed to explore both the abundance of glia cells and markers of their activation state to potentially unravel the intricate interplay between kynurenine metabolites and glia cells, and delineate their potential role as biomarkers, if found at circulant levels in the periphery.

Data obtained in the DLPFC gray matter of individuals affected by schizophrenia were partially unexpected. There was a trend to an increase KYNA levels and KYNA to QUINA ratio, which was consistent with previous findings and with the hypoglutamatergic hypothesis of schizophrenia (see above). However, we were surprised to find a significant incease in KYN, 3-HK, 3-HANA, and ANA levels, considering that 3-HK is formed by KMO, which is known to be defective in schizophrenia^11,34,67^. One possible explanation is that these metabolites accumulate because of a defective activity of 3-HAO, which converts 3-HANA into QUINA^11^. ANA is formed by KYN through a reaction catalyzed by kynureninase, and then hydroxylated into 3-HANA^20^. Interestingly, a 2-fold increase in ANA levels has been reported in the serum of patients affected by schizophrenia, and has been considered as a potential biomarker and treatment marker for schizophrenia^68^.

We have already reported that cinnabarinic acid is detectable at very low levels in the human DLPFC, is reduced in patients affected by schizophrenia, and this reduction did not correlate with age, sex, duration of the disease, and duration and type of antipsychotic medications^31^. Here we quantitated kynurenine metabolites in the gray and white matter of males and females and we showed that KYN, 3-HK, ANA, and 3-HANA are significantly increased both in the gray and white matter of the DLPFC of patients affected by schizophrenia. We run a thorough analysis correlating age and sex between controls and subjects affected by schizophrenia. The analysis revealed no significant differences in these demographic variables between the two cohorts, confirming the validity of our control group. The analysis run on male gray matter confirmed the increase of KYN, 3-HK, 3-HANA, and QUINA, whereas white matter showed an increase of all metabolites, but not XA. These significant increases were not found in female samples, suggesting that there could be a gender difference, although we have to note that the number of female samples are 4 in control subjects and 7 in the group of patients affected by schizophrenia. Moreover, we correlated kynurenine metabolite levels with age, duration of treatments (typical or atypical antipsychotic drugs) and neuropathology in patients affected by schizophrenia. We did not observe any correlation with these parameters, but a significant negative correlation between age and TRY and XA levels in the gray matter of DLPFC patients affected by schizophrenia, but not in control subjects. This is peripherally paralleled with the reported reduction of XA levels in the serum of patients affected by schizophrenia and their first-degree relatives^30^. It has been reported that activity of 3-hydroxyanthranilic acid dioxygenase (3-HAO), the enzyme that synthetizes QUINA, is significantly reduced in the DLPFC of schizophrenic patients. In line with this observation, we now report that 3-HANA levels are increased by about 3-fold in the gray matter, and about 4-fold in the white matter in the DLPFC of schizophrenic patients. This increase could be due to the accumulation of 3-HANA in response to the reduced activity of 3-HAO in patients affected by schizophrenia^11^.

In conclusion, this is the first report on measurements of all the metabolites of the kynurenine pathway in the DLPFC of control subjects and patients affected by schizophrenia. The results show increased levels of almost all kynurenine pathway metabolites suggesting a hyperactive pathway that could play a relevant role in the pathophysiology of schizophrenia. The parallelism between peripheral, as reported in the literature, and central levels of some metabolites could suggest their use as valuable predictive and prognostic biomarker candidates for schizophrenia^31^. In our experience, cinnabarinic acid is the only metabolite of all kynurenine metabolites to be reduced in the prefrontal cortex of individuals affected by schizophrenia^31^.

Our study has a number of limitations, including the presence of amyloid, tau or vascular patology in some samples of the CTRL and SCZ group, which might have caused local inflammation, thereby activating the kynurenine pathway. In addition, all samples from the SCZ groups were obtained from patients with a history of antipsychotic medication, which might have affected the activation of the kynurenine pathway. Finally, although there was no correlation between any of the kynurenine metabolite and the PMI, we cannot exclude that ante-mortem variables, such as the extent of the agonic state, duration of hypoxia, and the presence of fever might have influenced our findings.

## Materials and methods

### Human brain samples

Samples of the frontal lobe dorsolateral prefrontal cortex (DLPFC, Brodmann area 46, 9) from individuals affected by schizophrenia and non-schizophrenic controls were kindly provided by the Harvard Brain Tissue Resource Center, funded through NIH-NeuroBiobank HHSN-271-2013-00030C.

### UPLC/MS-MS analysis of kynurenine pathway metabolites

Measurements of kynurenine pathway metabolite levels were carried out in samples of DLFC of individuals affected by schizophrenia and non-schizophrenic controls. Detection and quantification of kynurenine pathway metabolites in tissue extracts were performed by ultra-performance liquid chromatography/tandem mass spectrometry (UPLC/MS/MS). Tissue extracts were prepared from about 40–50 mg of brain tissues, either white and gray matter, of patients affected by schizophrenia and healthy controls. White and gray matter tissues were sonicated in 0.1 N perchloric acid (weight/volume), homogenates were centrifuged at maximal speed in a microfuge for 30 min and supernatants were placed into vials for automatic injection into the UPLC system. The analysis was performed by the 1260 Infinity II Agilent Liquid Chromatography System separating molecules on a reversed-phase column (Poroshell 120, EC-C18, 1.9 µm, 2.1 × 50 mm - Agilent, Santa Clara, CA). Five μl were injected and the separation was obtained by a gradient using the eluent A (0.1% aqueous formic acid) and the eluent B (100% methanol) at a flow of 200 μl/min, using 10% solvent A for the first min and 100% solvent B for the following 3 min.

The mass spectrometry analysis was carried out on the 6470 LC/TQ Agilent triple quadrupole system equipped with a turbo ion spray source. The detector was set in the positive ion mode and the ion spray voltage was set at 5000 V (with a source temperature of 300 °C). Nitrogen was used as collision gas and the collision activation dissociation gas was set at medium value. A dynamic multiple reaction monitoring (dMRM) method was applied to detect and quantify kynurenine metabolites. The instrument was set in the dMRM mode, checking the transition *m/z* (in parenthesis collision energy) for TRY 204.7 → 172.7 (4); 204.7 → 76.9 (72); KYN 209 → 145.8 (20); 209 → 64.9 (56); ANA 138.2 → 119.9 (8); 138.2 → 64.9 (36); KYNA 190 → 143.9 (20); 190 → 88.8 (48); 3-HK 225.1 → 208 (8); 225.1 → 110 (16), XA 206.2 → 159.8 (20); 206.2 → 131.8 (36), 3-HANA 154.1 → 135.8 (12); 154.1 → 79.9 (32); QUINA 168.03 → 150 (8); 168.03 → 78 (24). Each analyte was monitored with a dwell time of 100 ms in the transitions from the precursor ion into the product ion and the mass spectrometer was tuned to obtain the best sensitivity for all transitions. The Mass Hunter software (Agilent, Santa Clara, CA) was used to analyze data. The calibration curve was tuned by dissolving different amounts of all kynurenine metabolites in acetonitrile and processing them in the identical way of tissue samples.

### Statistical analysis

Data were analyzed by Student’s *t* test. The Shapiro-Wilk normality test was run to met the criteria for normal distribution and the following Grubbs’s test was used to identify outliers. Correlation between kynurenine metabolite levels and different variables was carried out by linear regression analysis. Analyses were carried out by GraphPad software v. 8. The ANCOVA Analysis was carried out by IBM SPSS Statistics software v. 26.

### Supplementary information

Supp Figure Legends

Supplementary Figure 1

Supplementary Figure 2

Supplementary Figure 3

## Data Availability

The datasets generated and analysed during the current study are available in the repository NEUROMED.
