# Supplementary material for: Changes in kynurenine metabolites in the gray and white matter of the dorsolateral prefrontal cortex of individuals affected by schizophrenia
Source: Schizophrenia (Heidelb). 2024 Feb 27;10(1):27. doi: 10.1038/s41537-024-00447-3 (PMC10899223; doi:10.1038/s41537-024-00447-3)
Supplement: Supplementary file 1 — Supp Figure Legends [file 41537_2024_447_MOESM1_ESM.docx]

**SUPPLEMENTARY FIGURE LEGENDS**

**Figure 1S.** Statistical correlation analysis (simple linear regression) between postmortem interval (PMI) and levels of metabolites of the kynurenine pathway in the DLPFC of autoptic human brain samples. Grubbs’s test was performed once and one sample for each group was excluded as an outlier, when the case.

**Figure 2S.** Cumulative endogenous levels of kynurenine pathway metabolites in both grey and white matter of DLPFC in control subjects (n=24) and patients affected by schizophrenia (n=21) that did not reach statistical significance. Grubbs’s test was performed once and one sample for each group was excluded as an outlier, when the case.

**Figure 3S.** Correlation analysis between levels of kynurenine pathway metabolites in the grey and white matter and age or years of antipsychotic drug treatment. Values are as in Figure 2 and 2S.
